# Exploring the Underlying Mechanisms of Qingxing Granules Treating H1N1 Influenza Based on Network Pharmacology and Experimental Validation

**DOI:** 10.3390/ph17060731

**Published:** 2024-06-05

**Authors:** Hujun Du, Lianying Zhang, Haoxiang Sun, Shaoqin Zheng, Hongying Zhang, Shijia Yuan, Jiuyao Zhou, Zihao Fang, Jianping Song, Manxue Mei, Changsheng Deng

**Affiliations:** 1 Artemisinin Research Center, Guangzhou University of Chinese Medicine, Guangzhou 510405, China; duhujun620@outlook.com (H.D.); zhangjingshu13@outlook.com (L.Z.); shx227@foxmail.com (H.S.); zhhy0919@163.com (H.Z.); yuanshijia0521@163.com (S.Y.); songjp@gzucm.edu.cn (J.S.); 2 Sci-Tech Industrial Park, Guangzhou University of Chinese Medicine, Guangzhou 510330, China; zsqgz1213@163.com; 3 Department of Pharmacology, School of Pharmaceutical Sciences, Guangzhou University of Chinese Medicine, Guangzhou 510330, China; zhoujiuyao@tom.com; 4 The Eighth Clinical Medical School, Guangzhou University of Chinese Medicine, Guangzhou 510405, China; icarus-sky@outlook.com

**Keywords:** Qingxing granules, influenza A virus, H1N1, autophagy, network pharmacology, molecular docking, HPLC

## Abstract

Background: H1N1 is one of the major subtypes of influenza A virus (IAV) that causes seasonal influenza, posing a serious threat to human health. A traditional Chinese medicine combination called Qingxing granules (QX) is utilized clinically to treat epidemic influenza. However, its chemical components are complex, and the potential pharmacological mechanisms are still unknown. Methods: QX’s effective components were gathered from the TCMSP database based on two criteria: drug-likeness (DL ≥ 0.18) and oral bioavailability (OB ≥ 30%). SwissADME was used to predict potential targets of effective components, and Cytoscape was used to create a “Herb-Component-Target” network for QX. In addition, targets associated with H1N1 were gathered from the databases GeneCards, OMIM, and GEO. Targets associated with autophagy were retrieved from the KEGG, HAMdb, and HADb databases. Intersection targets for QX, H1N1 influenza, and autophagy were identified using Venn diagrams. Afterward, key targets were screened using Cytoscape’s protein–protein interaction networks built using the database STRING. Biological functions and signaling pathways of overlapping targets were observed through GO analysis and KEGG enrichment analysis. The main chemical components of QX were determined by high-performance liquid chromatography (HPLC), followed by molecular docking. Finally, the mechanism of QX in treating H1N1 was validated through animal experiments. Results: A total of 786 potential targets and 91 effective components of QX were identified. There were 5420 targets related to H1N1 and 821 autophagy-related targets. The intersection of all targets of QX, H1N1, and autophagy yielded 75 intersecting targets. Ultimately, 10 core targets were selected: BCL2, CASP3, NFKB1, MTOR, JUN, TNF, HSP90AA1, EGFR, HIF1A, and MAPK3. Identification of the main chemical components of QX by HPLC resulted in the separation of seven marker ingredients within 195 min, which are amygdalin, puerarin, baicalin, phillyrin, wogonoside, baicalein, and wogonin. Molecular docking results showed that BCL2, CASP3, NFKB1, and MTOR could bind well with the compounds. In animal studies, QX reduced the degenerative alterations in the lung tissue of H1N1-infected mice by upregulating the expression of p-mTOR/mTOR and p62 and downregulating the expression of LC3, which inhibited autophagy. Conclusions: According to this study’s network pharmacology analysis and experimental confirmation, QX may be able to treat H1N1 infection by regulating autophagy, lowering the expression of LC3, and increasing the expression of p62 and p-mTOR/mTOR.

## 1. Introduction

The influenza virus causes an acute respiratory infection known as influenza. Each year, there are around 1 billion cases of seasonal influenza, of which 3 to 5 million are severe cases [1]. There are four different varieties of influenza viruses: A, B, C, and D. Influenza A virus (IAV) is further subdivided into multiple subtypes, such as H1N1, H5N1, H3N2, and H7N9, based on the combination of surface proteins [2], with the H1N1 and H3N2 subtypes currently prevalent in humans. Increasing studies indicate that H1N1 infection can cause lung epithelial cell damage, leading to cytokine storms and severe acute pneumonia, and can also result in diseases like acute respiratory distress syndrome (ARDS), further increasing mortality and morbidity rates [3,4]. Due to the high variability of influenza, vaccines often do not provide sufficient protection; the main antiviral drugs used in clinics currently are neuraminidase inhibitors (such as oseltamivir) and M2 ion channel blockers (such as amantadine). Oseltamivir, the most widely used NA inhibitor globally, can cause adverse reactions like vomiting and body pain; M2 blockers have a high resistance rate and are no longer recommended clinically [5,6]. Thus, researching and developing effective and safe antiviral drugs is crucial.

Studies have shown that autophagy is involved in the pathogenesis of type A H1N1 influenza [7,8]. Autophagy, a conserved process in eukaryotic cells, involves the transport of substrates (such as damaged organelles, protein aggregates, and invading pathogens) from the cytoplasm to lysosomes under the regulation of autophagy-related genes (ATG), forming autolysosomes, where the substrates are degraded [9]. One of the pivotal players in the process of autophagy is the 1A/1B light chain 3 (LC3 or GABARAP), which p62 (Sequestosome 1, SQSTM1) can bind to, facilitating its assembly into autophagosomes. These autophagosomes subsequently merge with lysosomes to form autolysosomes, where the enclosed cargo, including ubiquitinated proteins, is degraded [10,11]. Research shows that H1N1 can induce the initiation of autophagy, increasing LC3 expression and leading to the accumulation of autophagosomes to facilitate virus replication [12,13,14]. Therefore, autophagy can be a focal point in researching drugs for treating H1N1.

Chinese medicine, with its advantages of high safety, multiple pathways, and multiple targets, is receiving increasing attention. Qingxing granules (QX), a traditional Chinese medicine formula used clinically for treating influenza, consists of ten ingredients: Artemisiae Annuae Herba, Armeniacae Semen Amarum, Bupleuri Radix, Scutellariae Radix, Forsythiae Fructus, Puerariae Lobatae Radix, Talcum, Mori Folium, Amomi Fructus Rotundus, and Poria. Studies indicate that baicalin from Scutellariae Radix can regulate the influenza virus protein NS1 through the PI3K/Akt signaling pathway, thereby exerting anti-influenza virus activity [15]. Compounds in Artemisiae Annuae Herba can alleviate inflammation caused by H1N1 infection through the TLR4/NF-κB (p65) signaling pathway [16]. Compounds in Bupleuri Radix can reduce H1N1 virus replication and pro-inflammatory cytokine production and improve pathological changes in lung tissues [17]. Preliminary studies suggest that Qingxing granules have antipyretic and analgesic effects and can treat dengue fever through the PERK/ATF6 pathway [18]. However, due to the complex components of QX, the underlying action mechanisms in treating H1N1 are unknown.

**Figure 1:**
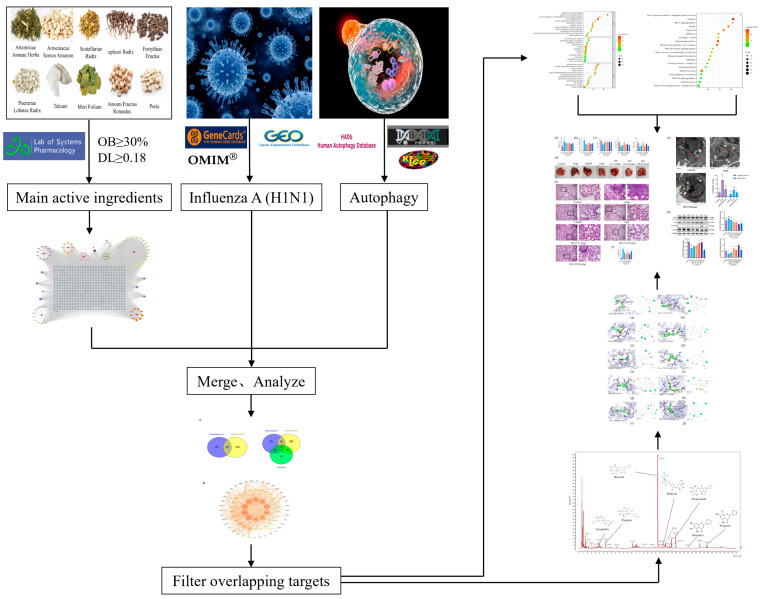
The technology roadmap of network pharmacology in this study.

Network pharmacology can create a “Compound-Protein/Gene-Disease” network, a rational approach for analyzing traditional Chinese medicine formulas [19]. This study established an in vivo “Component-Target” network for QX and used disease databases to determine H1N1 disease targets. We then built a PPI network for QX and explored key targets and pathways for QX treatment of H1N1 through online databases and enrichment analysis. Subsequently, we successfully established a mouse model of H1N1 infection and validated the potential mechanism of QX in treating type A H1N1 influenza in an H1N1-infected mouse model experiment. In order to meet our goals, we followed the technology roadmap outlined in Figure 1.

## 2. Results

### 2.1. Component Target Prediction of Qingxing Granules

Ninety-one candidate compounds were chosen from the TCMSP database based on the selection criteria of OB ≥ 30% and DL ≥ 0.18. Among these, 16 compounds were derived from Artemisia annua, 8 from bitter almond, 11 from Bupleurum, 28 from baicalin, 13 from Forsythia, 2 from Pueraria root, 16 from mulberry leaf, 9 from cardamom, and 1 from Poria. Following their extraction from the PubChem database, the canonical SMILES of the 91 compounds were loaded into SwissADME. A total of 786 possible targets of the QX effective components were found.

### 2.2. “Medicinal Material-Effective Component-Target” Network Map of Qingxing Granules

**Figure 2:**
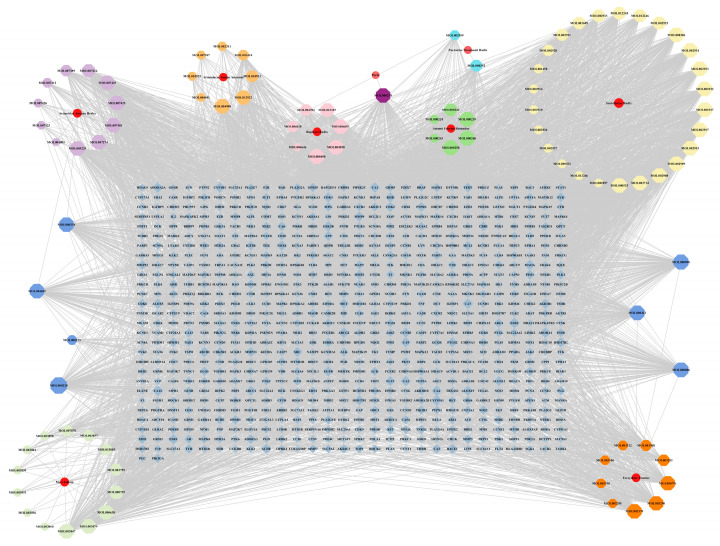
“Drug-Compound-Target” network of Qingxing granules.

A “Medicinal Material-Effective Component-Target” network map was created using Cytoscape 3.9.1 using the effective components and possible targets of QX, as seen in Figure 2. The network consists of 886 nodes and 5668 edges. Table 1 contains the specifics of the top 10 compounds, which were determined by their degree value. The 2D structures of the compounds were drawn using ChemDraw 20.0 software.

### 2.3. Prediction of Influenza A H1N1 Targets

Targets were collected from GenCards, OMIM, and GEO databases. Next, by filtering and normalizing the data in line with the Protein Database (Uniprot, https://www.uniprot.org/, accessed on 18 December 2023) [20], duplicates were removed from all targets. Regarding influenza A H1N1, 5420 targets were acquired.

### 2.4. Prediction of Autophagy-Related Genes

Autophagy-related genes were selected from the KEGG, HADb, and HAMdb databases. Following the removal of duplicate genes, 821 autophagy-related targets were found.

### 2.5. Prediction of Qingxing Granules PPI Network

**Figure 3:**
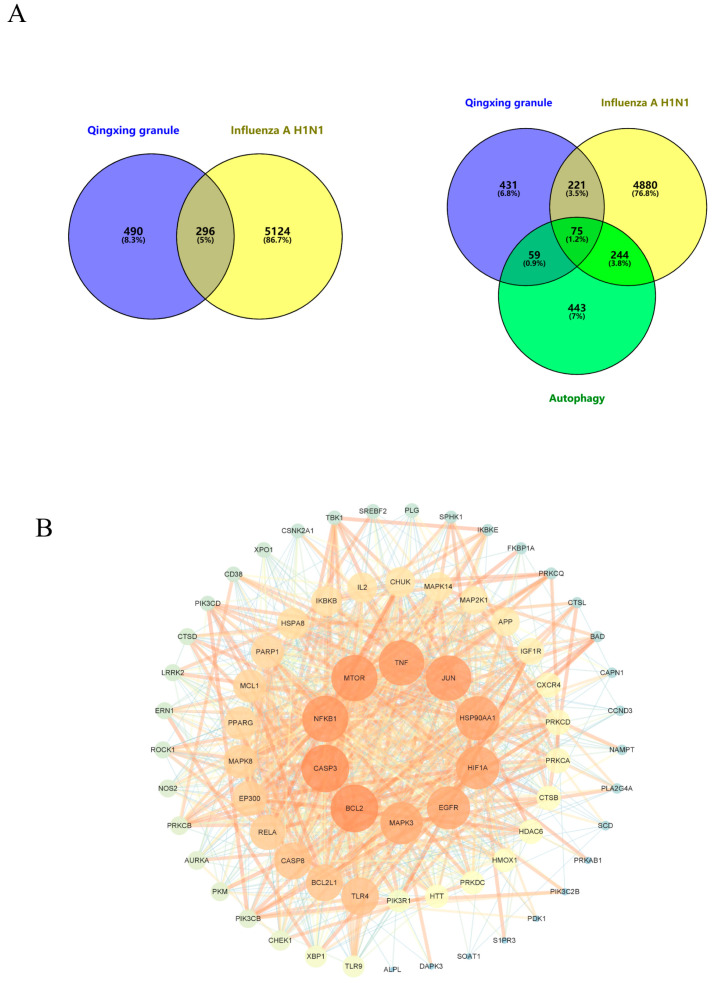
Core target screening of Qingxing granules. (**A**) Venn diagrams of QX “Component Target-Disease Target” and “Component Target-Disease Target-Autophagy Gene”; (**B**) PPI network of QX treatment for H1N1.

Figure 3A shows a Venn diagram illustrating the junction of disease targets, autophagy-related targets, and probable targets of QX’s effective components. The diagram shows 296 common targets between QX’s potential targets and disease targets and 75 common targets among QX’s potential targets, disease targets, and autophagy-related targets. These 75 intersecting targets were inputted into STRING 12.0, and a PPI network map of QX treatment for influenza A H1N1 was constructed using Cytoscape 3.9.1 (Figure 3B). The network contains 73 nodes and 919 edges. Analysis of the network revealed an average node degree of 25.178, with 32 targets higher than the average. The top 10 core targets were: BCL2, CASP3, NFKB1, MTOR, JUN, TNF, HSP90AA1, EGFR, HIF1A, and MAPK3 (details of core targets in Table 2).

### 2.6. GO Enrichment Analysis

**Figure 4:**
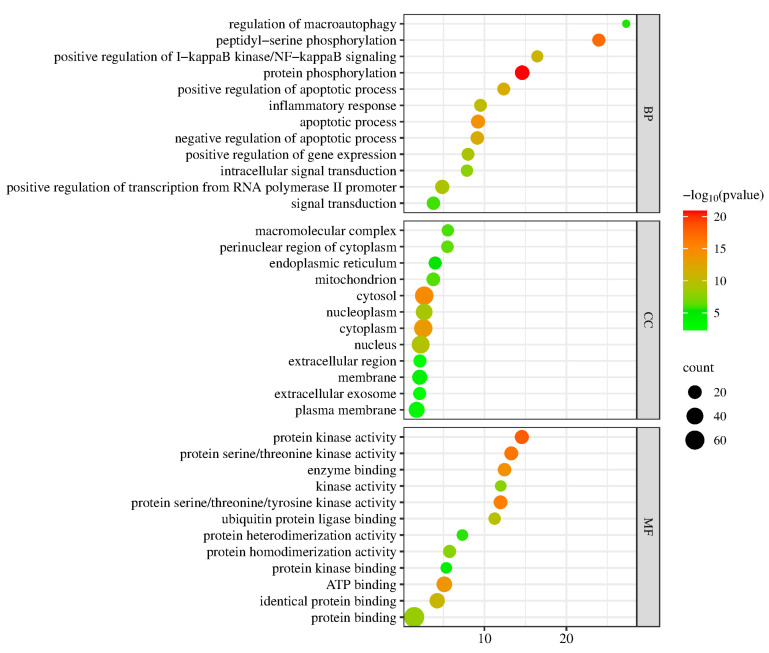
GO enrichment analysis bubble chart.

We used the DAVID database for the GO analysis of targets in the PPI network. The gene ratio (%) was used to pick the top 10 GO keywords (*p* < 0.05) for BP, CC, and MF data in descending order of magnitude. The top five BPs include peptidyl-serine phosphorylation, protein phosphorylation, positive regulation of NF-kappaB signaling, regulation of macroautophagy, and positive regulation of the apoptotic process, as illustrated in Figure 4. The macromolecular complex, cytosol, endoplasmic reticulum, mitochondrion, and perinuclear area of cytoplasm are the top five CCs. Enzyme binding, kinase activity, protein kinase activity, protein serine/threonine/tyrosine kinase activity, and protein kinase activity are the top five MFs.

### 2.7. KEGG Pathway Enrichment Analysis

**Figure 5:**
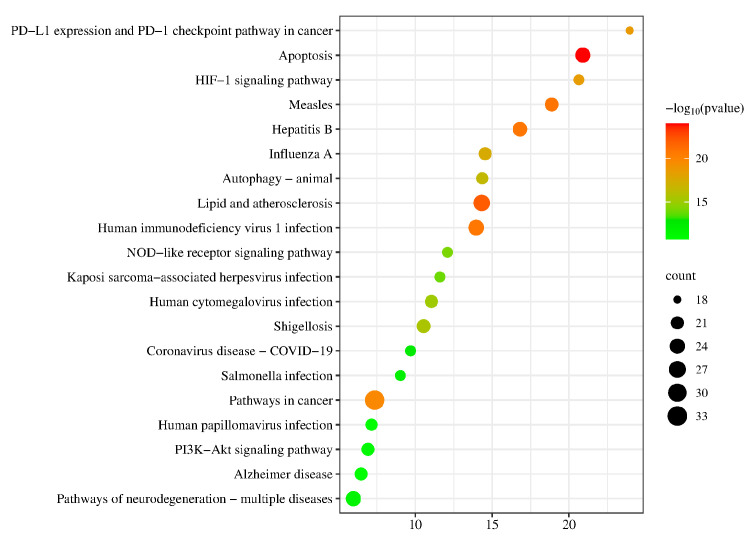
KEGG pathway enrichment analysis bubble chart.

We used the DAVID database to do a KEGG pathway enrichment analysis on the 75 candidate targets. Based on the gene ratio (%) in descending order, we identified the top 20 pathways (*p* < 0.05). Measles, hepatitis B, apoptosis, PD-L1 expression and the PD-1 checkpoint pathway in cancer, and the HIF-1 signaling pathway are the top five pathways shown in Figure 5.

### 2.8. Determination of the Main Chemical Components in QX

**Figure 6:**
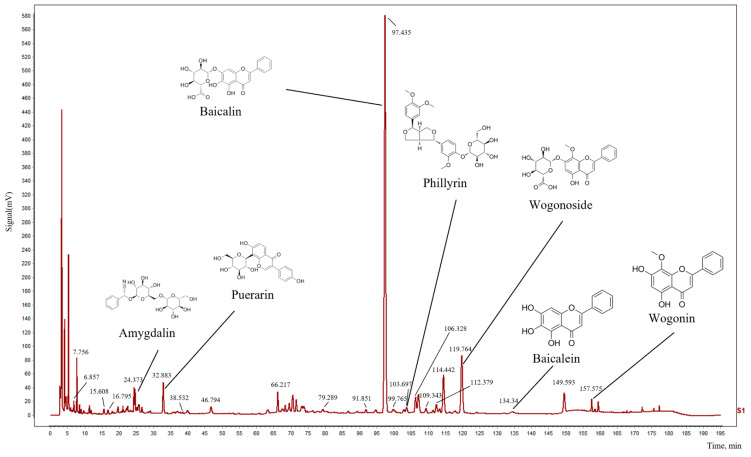
HPLC characteristic chromatogram of Qingxing granules.

As shown in Figure 6 and Table 3, seven marked components were separated within 195 min. The retention times for amygdalin, puerarin, baicalin, phillyrin, wogonoside, baicalein, and wogonin were as follows: 24.373, 32.883, 97.435, 103.697, 119.764, 134.340, and 157.575 min, respectively.

### 2.9. Molecular Docking

**Figure 7:**
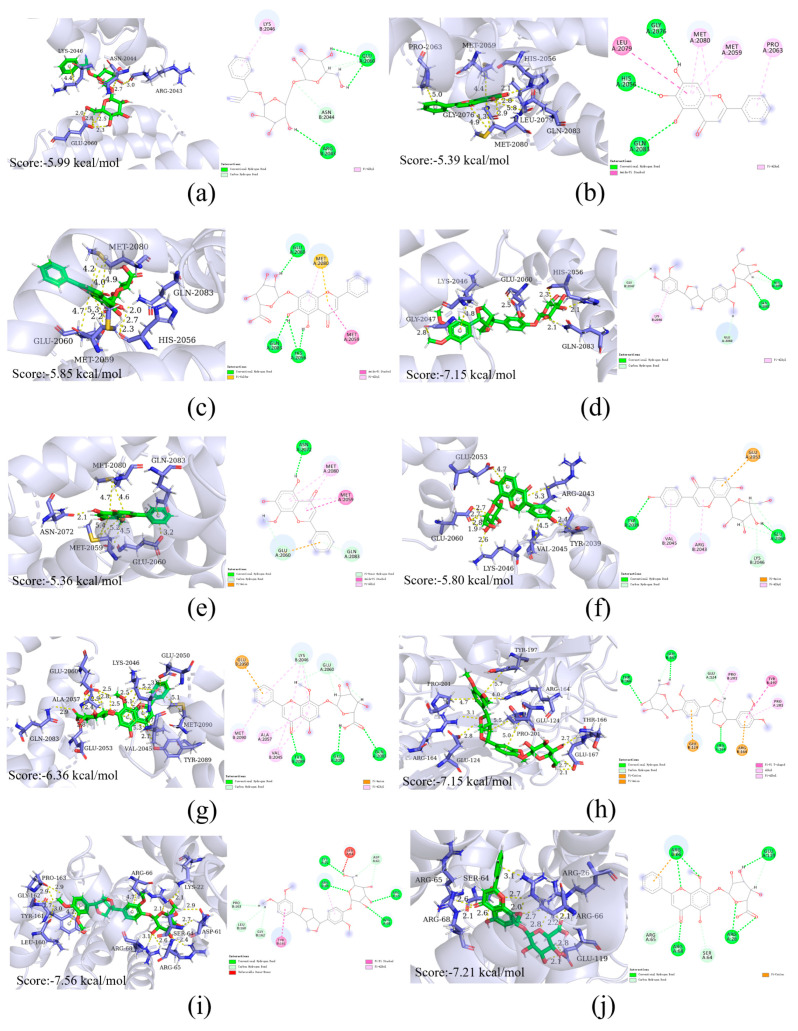
Molecular docking models of key compounds and core targets. (**a**) AmygdalinmTOR; (**b**) baicalein-mTOR; (**c**) baicalin-mTOR; (**d**) phillyrin-mTOR; (**e**) puerarin-mTOR; (**f**) wogonin-mTOR; (**g**) wogonoside-mTOR; (**h**) phillyrin-CASP3; (**i**) phillyrin-BCL2; (**j**) wogonoside-BCL2.

We obtained the protein structures corresponding to the top four core targets (BCL2, CASP3, NFKB1, MTOR) from the PDB database, and selected the top three compounds in Qingxing granules (details in Table 4) and seven compounds from HPLC results for molecular docking. Using AutoDock Vina 1.2.0, we input 40 sets of target proteins and compound small molecules, calculating the affinity values for each binding site (results in Table 3). A binding energy of less than −5 indicates good binding activity between the ligand and receptor. Among the 40 sets, 39 sets were successfully bound, with 38 sets having affinity values less than −5 kcal/mol. We have displayed some of the molecular docking results in Figure 7.

### 2.10. Improved Effects of QX on Mice Infected with H1N1

**Figure 8:**
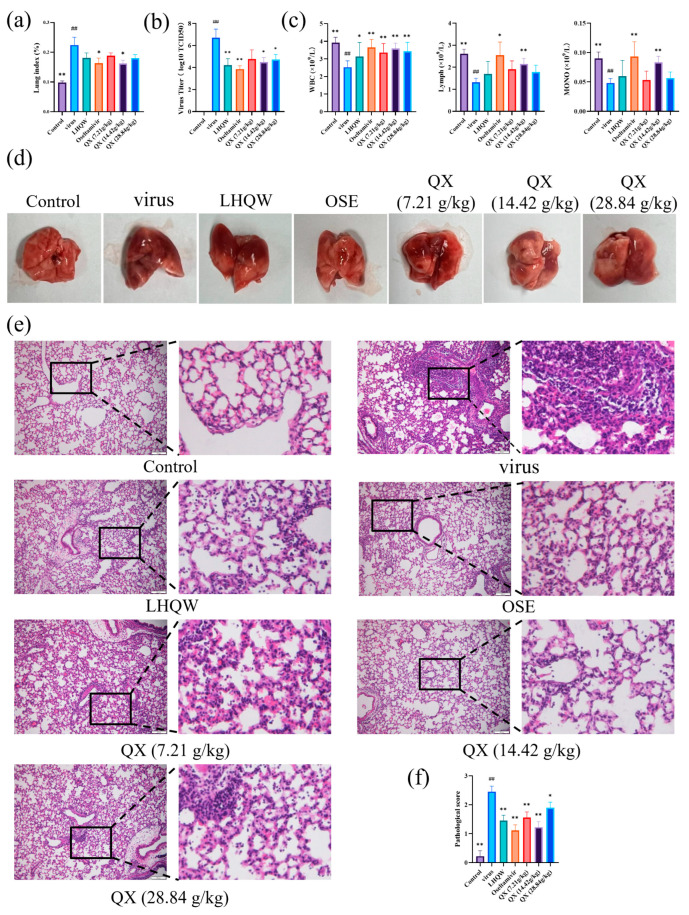
Improvement effect of QX on mice infected with H1N1. (**a**) Changes in lung index of different groups of mice; (**b**) changes in H1N1 virus titers in lung tissues of mice from different groups; (**c**) variations in the overall white blood cell, lymphocyte, and monocyte characteristics in mouse blood; (**d**) the gross appearance of lung tissues from different groups of mice; (**e**) HE staining observations of mice in different groups. Scale bar = 100 µm; (**f**) The lung tissue was assessed using a histopathological grading scale, which ranged from 0 (indicating no alterations) to 3 (representing severe pathological conditions). Data are presented as mean ± SD. * *p* < 0.05, ** *p* < 0.01, compared with the virus group; ^##^ *p* < 0.01, compared with the Control group.

To explore the effect of QX on mice infected with H1N1, we conducted an in vivo animal experiment. As shown in Figure 8a, the lung index of the virus group was 0.22 ± 0.026, significantly higher than the control group’s (0.098 ± 0.005, *p* < 0.01) value. In contrast to the virus group, the medium-dose QX group (14.42 g/kg) and the oseltamivir group showed improved lung indices (0.162 ± 0.011 and 0.163 ± 0.017, respectively) (*p* < 0.05). The lung indices of the Lianhua Qingwen (LHQW), low-dose QX group (7.21 g/kg), and high-dose QX group (28.84 g/kg) were lower compared to the infection group, with lung indices of 0.181 ± 0.016, 0.188 ± 0.009, and 0.18 ± 0.012, respectively. The results of H1N1 virus titration in the lung tissues of mice are shown in Figure 8b. Compared to the normal group, the H1N1 virus significantly increased in the lung tissues of the virus group (*p* < 0.01). Compared to the virus group, the lung tissue H1N1 viral loads decreased in the LHQW group, OSE group, QX medium-dose group (14.42 g/kg), and QX high-dose group (28.84 g/kg), with the exception of the QX low-dose group (7.21 g/kg) (*p* < 0.01; *p* < 0.01; *p* < 0.05; *p* < 0.05). As shown in Figure 8c, we compared blood parameters among different groups of mice. Blood samples were collected on the 6th day after infection, examining alterations in monocytes, lymphocytes, and white blood cells. When compared to the normal control group, the total white blood cells, lymphocytes, and monocytes in the virus group significantly decreased (*p* < 0.01; *p* < 0.01; *p* < 0.01). The total counts of white blood cells, lymphocytes, and monocytes increased when the QX medium dose (14.42 g/kg) was administered (*p* < 0.01; *p* < 0.01; *p* < 0.01). The gross morphology of mouse lung tissues is shown in Figure 8d, where lung tissues of the virus group were slightly larger in volume, darker in color, and showed mostly congested and edematous solidified areas. The areas of solidification in the OSE and medium-dose QX group mice were reduced. The histopathological sections of the lung tissues stained with HE are shown in Figure 8e. The virus group showed severe lung damage, developing into interstitial pneumonia, characterized by a large number of collapsed alveoli, thickened alveolar walls, severe inflammatory infiltration around the bronchi, and bleeding. Compared to the virus group, the alveolar collapse in the medium-dose QX (14.42 g/kg), LHQW, and OSE groups was lighter, and there was some inhibition of the development of interstitial pneumonia, with improved congestion in alveolar walls and interstitial vessels.

### 2.11. Transmission Electron Microscopy

**Figure 9:**
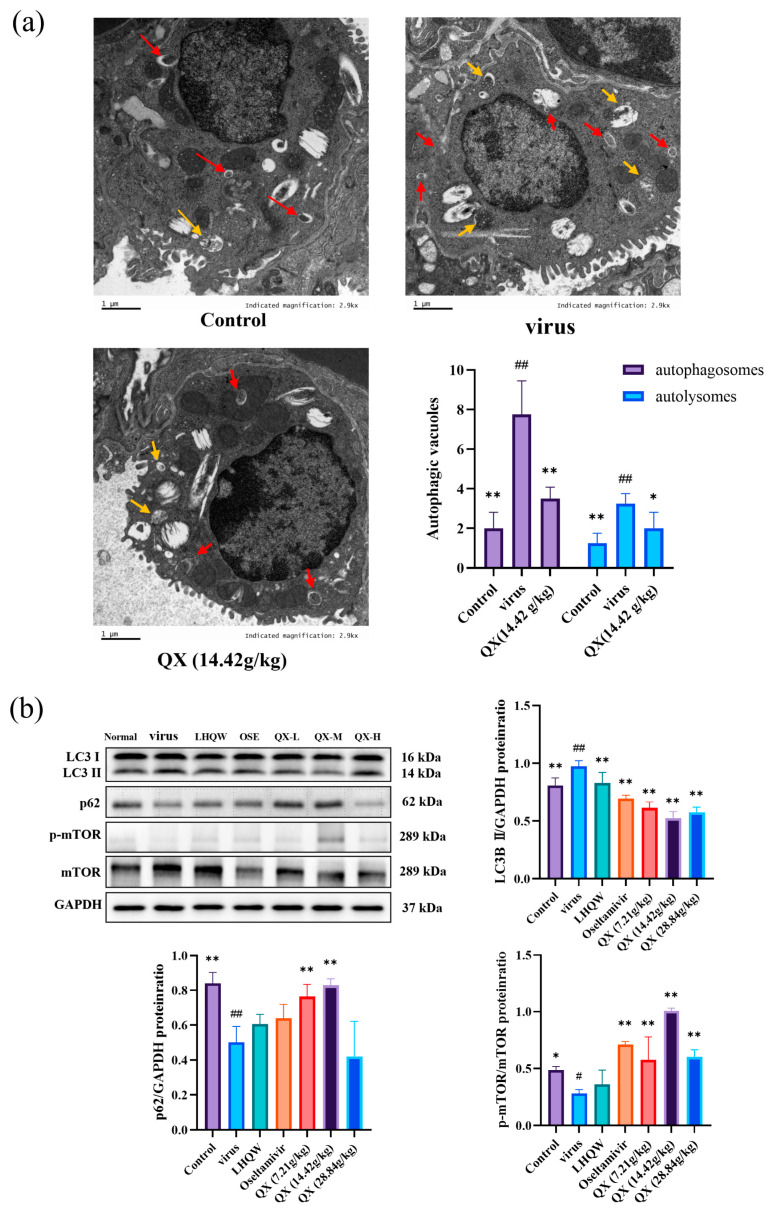
Qingxing granules affect mice infected with H1N1 through autophagy. (**a**) Observation of the number of autophagosomes and autolysosomes in the normal group, virus group, and medium-dose QX group mice under transmission electron microscopy. The red arrow points to the target as the autophagosome. The yellow arrow points to the autolysosome. Scale bar = 1 µm; (**b**) LC3, p62, and p-mTOR/mTOR expression measurements in mouse lung tissue employing Western blot research. Data are presented as mean ± SD. * *p* < 0.05, ** *p* < 0.01, compared with the virus group; ^#^ *p* < 0.05, ^##^ *p* < 0.01, compared with the Control group.

The effect of the medium dose of Qingxing granules (14.42 g/kg) on the number of autophagosomes and autolysosomes in the lung epithelial cells of mice infected with H1N1 was observed under a transmission electron microscope. Figure 9a shows that the model group mice’s lung tissue had a significantly higher number of autophagosomes and autolysosomes than the normal group’s. In contrast, the medium dose group (14.42 g/kg) of QX significantly reduced the number of autophagosomes and autolysosomes in the lung tissue, indicating that the medium dose of QX (14.42 g/kg) can inhibit the formation of autophagosomes and autolysosomes in mice infected with H1N1.

### 2.12. Western Blot

Western blot studies were used to determine the levels of protein expression for LC3, p62, p-mTOR, and mTOR. As can be shown in Figure 9b, the virus group had higher (*p* < 0.01) expression levels of LC3-II and lower (*p* < 0.01; *p* < 0.05) expression levels of p62 and p-mTOR/mTOR than the normal group. Following QX treatment, p62 and p-mTOR/mTOR expression rose, whereas LC3-II expression levels were suppressed. The amounts of LC3-II in the QX low, medium, and high dose groups all dramatically dropped (*p* < 0.01; *p* < 0.01; *p* < 0.01). After receiving the low and medium dose groups, the level of p62 increased (*p* < 0.01; *p* < 0.01). Following treatment with the low, medium, and high dose groups of QX, the expression of p-mTOR/mTOR significantly increased (*p* < 0.01; *p* < 0.01; *p* < 0.01).

## 3. Discussion

Influenza A virus (IAV) is an acute infectious respiratory disease that poses a significant threat to human society, severely affecting public health and the economy. The necessity for research and development of safe and effective antiviral treatments is underscored by the potential of antiviral drugs, such as amantadine and oseltamivir, to produce drug resistance [21,22]. Traditional Chinese medicine (TCM) offers advantages in treating H1N1 infection such as multi-target effects, low resistance, and high safety. Notably, autophagy is involved in the disease process of H1N1; the virus can activate autophagy initiation, increase autophagosome formation, and simultaneously block autophagosome–lysosome fusion to inhibit the autophagic degradation pathway, promoting viral replication [23]. Consequently, in this work, we employed network pharmacology to forecast QX’s targets for treating H1N1, and we corroborated these results through animal studies.

Using network pharmacology, we constructed a target network, identifying 91 effective components of QX and selecting the 10 most relevant compounds based on the network, including quercetin, kaempferol, and isorhamnetin, which have been experimentally proven to treat H1N1. Quercetin has been shown by Bipin Vaidya et al. to inhibit H1N1 virus multiplication by controlling the production of important proteins such as heat shock proteins, fibronectin 1, and prohibitin [24]. Neuraminidase (NA), a glycoprotein on the surface of the influenza virus, facilitates viral movement in the respiratory tract by catalyzing the cleavage of sialic acid residues [25]. Ai-Lin Liu et al. discovered that kaempferol can inhibit H1N1 by suppressing the NA protein [26]. During autophagy, the protein LC3, which is known as an autophagy marker and is involved in the process, changes from cytoplasmic LC3-I to membrane-bound LC3-II. LC3-II is widely used to quantify autophagic activity, reflecting the number of autophagosomes [27]. Ahmed Abdal Dayem et al. found that isorhamnetin could inhibit virus-induced autophagy by suppressing the lipidation of LC3-II [28].

In summary, the effective components of QX likely exert their therapeutic effects on H1N1 through synergistic relationships. Based on the results of the PPI network analysis, we identified the ten most significant potential targets of QX in the treatment of H1N1 as follows: BCL2, CASP3, NFKB1, MTOR, JUN, TNF, HSP90AA1, EGFR, HIF1A, and MAPK3. We then deduced that QX might treat H1N1 via pathways such as the PD-1 checkpoint pathway, apoptosis, HIF-1 signaling route, autophagy, and NOD-like receptor signaling pathway based on the findings of GO and KEGG enrichment analyses.

Research conducted on animals has demonstrated that QX can ameliorate pathological abnormalities in the lung tissues of infected mice, reduce the lung index and the viral titer of H1N1 in lung tissue, and undo modifications in the total white blood cells, lymphocytes, and monocytes in the blood of infected mice. Using electron microscopy, we were able to detect an increase in autophagosomes in the lung tissues of H1N1-infected mice, which subsequently decreased following treatment with a modest dose of QX. A key regulator of cellular metabolism, mTOR is a serine/threonine kinase. Autophagy is triggered when the mTOR signaling pathway is inhibited [29]. Western blot analysis revealed that following H1N1 infection, mouse lung tissue displayed increased expression of the proteins LC3-II, but p62 and p-mTOR/mTOR levels declined. Following QX treatment, p-mTOR/mTOR and p62 expression were increased, whereas LC3-II protein expression was decreased. From these findings, we conclude that H1N1 infection causes pathological damage in mouse lung tissue and can inhibit the mTOR pathway, inducing autophagy and leading to the accumulation of autophagosomes. In mice infected with H1N1, QX can ameliorate the pathological alterations in the lung tissues, stimulate the mTOR pathway, and inhibit autophagy.

## 4. Materials and Methods

### 4.1. Screening of Active Biological Compounds and Related Targets in Qingxing Granules

The Traditional Chinese Medicine Systems Pharmacology Database (TCMSP, https://old.tcmsp-e.com/tcmsp.php, accessed on 10 December 2023) [30] was used to conduct a targeted search of compounds from the ten constituent herbs of QX. We searched for the active and effective ingredients in QX based on oral bioavailability (OB) ≥ 30% and drug-likeness (DL) ≥ 0.18 [31]. After scanning the PubChem database (https://pubchem.ncbi.nlm.nih.gov, accessed on 10 December 2023) [32] for the pertinent SMILES for the active components, they were entered into SwissADME (http://www.swissadme.ch/, accessed on 12 December 2023) [33]. Based on gastrointestinal absorption (GI absorption) and Lipinski’s rule of five, we obtained the targets of the effective components. The final deduplicated targets were identified as the related targets of the effective components of Qingxing granules.

### 4.2. Network Construction of “Medicinal Material-Active Component-Target” of Qingxing Granules

Using Cytoscape software (version 3.9.1) [34], we established and visualized the “Medicinal Material-Active Component-Target” network. In the network, nodes represent medicinal materials, active components, and targets, while edges represent their interactions.

### 4.3. Collection of Targets Related to Influenza A H1N1

We gathered targets associated with the H1N1 sickness from the GeneCards (https://www.genecards.org/, accessed on 13 December 2023) [35], OMIM (https://omim.org/, accessed on 13 December 2023) [36], and GEO (https://www.ncbi.nlm.nih.gov/geo/, accessed on 13 December 2023) [37] (dataset ID: GSE21802 [38]) databases and used the keyword “Influenza A H1N1” to find them. We then merged all targets from these three databases and removed duplicates to obtain the final set of H1N1-related targets.

### 4.4. Collection of Autophagy-Related Genes

The Human Autophagy Database (HADb, http://www.autophagy.lu, accessed on 15 December 2023) [39], the Kyoto Encyclopedia of Genes and Genomes Database (KEGG, https://www.kegg.jp/, accessed on 15 December 2023) [40], and the Human Autophagy Modulator Database (HAMdb, http://hamdb.scbdd.com/home/index/, accessed on 15 December 2023) [41] were the sources of the genes linked to autophagy. To obtain targets relevant to autophagy genes, we combined and removed duplicates from these three databases.

### 4.5. Construction and Analysis of the Protein–Protein Interaction (PPI) Network

The important targets of the Qingxing granules’ effective components, H1N1’s sickness targets, and autophagy-related targets were uploaded to the Venny database (version 2.1, https://bioinfogp.cnb.csic.es/tools/venny/, accessed on 15 December 2023) in order to get intersecting genes. After that, these intersecting genes were saved as a “tsv” format file and loaded into the STRING database (version 12.0, https://cn.string-db.org/, accessed on 15 December 2023) [42] with “Homo sapiens” set as a limitation. The software Cytoscape was then used to import this information and analyze it in order to create a protein–protein interaction (PPI) network.

### 4.6. GO Enrichment Analysis and KEGG Pathway Enrichment Analysis

KEGG pathway analysis and GO functional annotation were performed using the DAVID database (version 6.8, https://david.ncifcrf.gov/, accessed on 25 December 2023) [43]. Following the input of the target gene names, the species was restricted to “humans”, and each target gene name was modified to correspond with its official gene symbol. The selection criteria were based on *p*-values, with the top 10 in biological processes (BP), cellular components (CC), and molecular functions (MF) selected for GO analysis; the top 20 entries were selected for KEGG pathway analysis. Finally, using the Weishengxin platform (https://www.bioinformatics.com.cn/, accessed on 25 December 2023), bubble charts for GO analysis and KEGG pathway analysis were made in order to observe the biological relevance of the shared targets of Qingxing granules and H1N1.

### 4.7. Medicinal Herbs

Guangzhou Hancao Pharmaceutical Technology Co., Ltd. (Guangzhou, China) supplied Artemisiae Annuae Herba (batch number: 191001), Scutellariae Radix (batch number: 200302), Puerariae Lobatae Radix (batch number: 191201), Forsythiae Fructus (batch number: 200201), Mori Folium (batch number: 200301), Bupleuri Radix (batch number: 200201), Amomi Fructus Rotundus (batch number: 191001), and Poria (batch number: 200401). Guangzhou Zhixin Chinese Herbal Medicine Co., Ltd. (Guangzhou, China) supplied Armeniacae Semen Amarum (batch number: 200201) and Talcum (batch number: 200201). All the above herbs have been identified by Chief Pharmacist Xiaohong Yuan from the Guangdong Provincial Hospital of Traditional Chinese Medicine.

### 4.8. Preparation Process of Qingxing Granules

The formula ratio of QX (batch number:20201028) is as follows: Artemisia annua: bitter almond: Bupleurum: baicalin: Forsythia: Pueraria root: Talc: mulberry leaf: cardamom: Poria (5:5:5:5:5:5:3:5:5:5). These ten kinds of medicinal slices were weighed in proportion, 10 times the volume of water was added, the mixture was soaked for 30 min, and decocted twice, each for 2 h. The decoction (400 mesh) was filtered and the filtrate was concentrated under reduced pressure at 80 °C (vacuum degree −0.090 to −0.1 MPa) until the relative density reached 1.05 to 1.15 (60–70 °C) to obtain a concentrated extract. Then, the product was vacuum dried at 70 °C to obtain a dry paste-like powder, used as crude medicine. The details of the medicinal materials of Qingxing granules are provided in Table 5.

### 4.9. HPLC Analysis of Qingxing Granules

The main chemical components of QX were identified through high-performance liquid chromatography (HPLC). Chromatographic separation was carried out using a high-performance liquid chromatograph (Shimadzu LC-20AT) (Shimadzu Corporation, Kyoto, Japan), equipped with an Agela Technologies Venusil ASB C18 column (5 μm, 4.6 mm × 250 mm). The reference standards are as follows: baicalin (batch number: B20570, purity: 98%), baicalein (batch number: B20571, purity: 98%), wogonoside (batch number: B20488, purity: 98%), wogonin (batch number: B20489, purity: 98%), phillyrin (batch number: B20725, purity: 98%), amygdalin (batch number: B20687, purity: 98%), and puerarin (batch number: B20446, purity: 98%). All reference standards were purchased from Shanghai Yuanye Bio-Technology Co., Ltd., Shanghai, China. The mobile phase consisted of acetonitrile (A) and 0.1% phosphoric acid solution (B). Gradient elution was performed according to the conditions specified in Table 6. The flow rate was 1.0 mL/min, the column temperature was 30 °C, the detection wavelength was 210 nm, and the injection volume was 10 μL.

### 4.10. Molecular Docking

The Traditional Chinese Medicine Systems Pharmacology Database (TCMSP) had the top 3 chemicals, which were listed as mol2 format files and sorted by degree value in descending order. The compounds from the HPLC results were added as mol2 format files. The top four core target proteins identified from the PPI network analysis were entered into the Protein Database (PDB, http://www.rcsb.org, accessed on 25 December 2023) [44] to determine their structures and download them. The AutoDock Vina software (Version 1.2.0) was then used to perform molecular docking of the compound small molecules with the key target proteins. Finally, PyMOL (version 2.4) was used to illustrate the optimal combinations of docking scores.

### 4.11. Animals and Viruses

Specific pathogen-free (SPF) female BALB/c mice, weighing 12 ± 2 g, provided by Guangzhou Southern Medical University Laboratory Animal Science and Technology Development Co., Ltd. (Guangzhou, China; production license: SCXK (Yue) 2021-0041) were adaptively bred for a week at a temperature of 20–25 °C with unrestricted access to food and drink. All animal research at Guangzhou University of Chinese Medicine Science and Technology Industrial Park has been approved by the Experimental Animal Management and Use Committee (approval number: PZ23096).

H1N1 (influenza virus A/PR/8/34, PR8) was provided by the Guangdong Provincial Key Laboratory of New Drug Screening at Southern Medical University. The virus strain was stored at −80 °C.

### 4.12. Animal Model

The mouse model infected with H1N1 was developed in the manner previously outlined [45]. The mice were randomly assigned to seven groups after a week of adaptive feeding: Lianhua Qingwen (LHQW), oseltamivir (OSE), blank control, model control, low-dose, medium-dose, and high-dose Qingxing granules groups. All mice, except those in the blank control group, were intraperitoneally injected with 1% pentobarbital sodium (0.1 mL/10 g) and intranasally administered 50 μL of virus solution (H1N1) (LD_50_ = 10^−3.85^/50 μL [46]), while the blank control group received an equal volume of PBS. According to the “Pharmacological Research Methods of Traditional Chinese Medicine”, the drug gavage dosage for mice was calculated based on the body surface area conversion between humans and mice. The blank group mice were given an equal volume of saline by gavage every day, and the infected group mice were given QX (7.21, 14.42, 28.84 g/kg/day), oseltamivir (St. Louis, MO, USA), or Lianhua Qingwen (batch number: B2111137) (YILING, Shijiazhuang, China) by gavage every day for five consecutive days. On the sixth day, the mice were intraperitoneally injected with 1% pentobarbital sodium (0.1 mL/10 g) for anesthesia. Following anesthesia, orbital blood collection was performed on the mice. Subsequently, the mice were euthanized by cervical dislocation to obtain lung tissues, and the lung index was recorded.

### 4.13. Routine Blood Test

Mouse blood was collected and counted using an automatic blood analyzer (ADVIA 2120i, SIEMENS, Erlangen, Germany) to observe white blood cells, lymphocytes, and monocytes.

The viral load of the H1N1 influenza virus in mouse lung tissues was detected using the cytopathic effect method. The right lower lobes of the lungs were harvested for the assay. After grinding the lung tissues, they were centrifuged to obtain the viral supernatant. The viral supernatant was diluted in a 10-fold gradient and added to MDCK cells to observe and record the cytopathic holes.

### 4.14. Hematoxylin and Eosin (HE) Staining

The lung tissues of mice were embedded in paraffin after being treated with 4% paraformaldehyde. After dividing each tissue into 4 μm sections, the pathological changes were examined and assessed using a microscope (BX63, Olympus Corporation, Tokyo, Japan) after each section had been stained with hematoxylin and eosin. Pathological scoring of the degree of damage to lung tissue is rated on a scale of 0 to 3 [47].

### 4.15. Transmission Electron Microscopy

After being fixed for 24 h in 2.5% glutaraldehyde, fresh lung tissue was washed three times with PBS for 10 min at a time. Subsequently, the tissue was fixed in 1% osmium tetroxide for 2 h, followed by three washes with PBS, each for 10 min. The samples then underwent a series of dehydration steps with ethanol and acetone, followed by acetone soaking. After each sample was embedded, an ultramicrotome was used to create ultrathin slices with a thickness of about 100 nm. After that, the sections were stained with lead citrate and uranyl acetate and then examined using a transmission electron microscope (Tecnai G2 Spirit, FEI Company (Hillsboro, OR, USA)) to observe the number of autophagosomes and autolysosomes, as well as the ultrastructure of the alveolar epithelial cells in H1N1 model rats.

### 4.16. Western Blot Analysis

Western blot was used to assess the expression levels of mTOR, p-mTOR, LC3, and p62 proteins in lung tissues. After lung tissue samples were gathered, lung tissue protein extracts were made in phosphatase and protease inhibitor-containing RIPA lysis buffer. Protein was separated using 10% (#PG112) and 12.5% (#PG113) PAGE gels (EpiZyme, Shanghai, China) and subsequently placed onto PVDF membranes in equal amounts. The membranes were blocked for one hour at room temperature using a fast-blocking solution (#PS108P), and then they were incubated with primary antibodies against mTOR (#2983S) (1:1000), p-mTOR (#2971S) (1:1000), p62 (#23214S) (1:1000), and LC3A/B (#12741S) (1:1000) for an entire night at 4 °C. These antibodies were purchased from Cell Signaling Technology (Danfoss, MA, USA), and GAPDH (#LF206) (1:3000) was purchased from Epizyme (Shanghai, China). After three five-minute washes in Tris-buffered saline with Tween-20 (TBST), the membranes were incubated for an hour at room temperature with HRP-conjugated secondary antibody goat anti-rabbit IgG (#LF102) (EpiZyme, Shanghai, China). Using a SuperSignal chemiluminescent substrate kit (#SQ201L) (EpiZyme, Shanghai, China), the membranes were detected after being rinsed five more times with TBST, each for five minutes. ImageJ software version 1.48 was utilized for quantitative analysis of protein bands.

### 4.17. Statistical Analysis

Version 27.0 of SPSS was used to analyze all of the study’s data. Standard deviation (S.D.) ± mean is how the data are displayed. ANOVA in one direction was used to examine group differences. *p*-values less than 0.05 were considered significant in statistics.

## 5. Conclusions

The findings of our investigation have demonstrated that one important mechanism by which QX may protect against H1N1 is via activating the mTOR signaling pathway, indicating that QX is a promising drug to treat H1N1.

## Figures and Tables

**Table 1:** The specifics of the top 10 compounds.

Mol ID	Compound Name	Molecular Function	2D Structure	Degree	BC	CC	ASPL
MOL000228	(2R)-7-hydroxy-5-methoxy-2-phenylchroman-4-one	C_16_H_14_O_4_	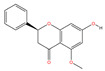	131	0.0628	0.3839	2.6045
MOL000098	Quercetin	C_15_H_10_O_7_	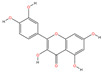	108	0.0093	0.3767	2.6542
MOL000258	Dehydrodiisoeugenol	C_19_H_20_O_4_	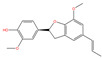	108	0.0513	0.3751	2.6655
MOL000006	Luteolin	C_15_H_10_O_6_	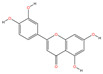	106	0.0087	0.3748	2.6677
MOL000260	5-[(2R,3R)-7-methoxy-3-methyl-5-[(E)-prop-1-enyl]-2,3-dihydrobenzofuran-2-yl]-1,3-benzodioxole	C_20_H_20_O_4_	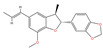	105	0.0777	0.3729	2.6813
MOL000239	Jaranol	C_17_H_14_O_6_	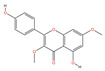	104	0.0106	0.3745	2.6700
MOL000422	Kaempferol	C_15_H_10_O_6_	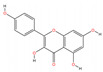	104	0.0078	0.3754	2.6632
MOL000354	Isorhamnetin	C_16_H_12_O_7_	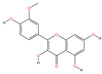	102	0.0069	0.3732	2.6790
MOL004609	Areapillin	C_18_H_16_O_8_	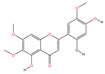	102	0.0068	0.3729	2.6813
MOL000490	Petunidin	C_16_H_13_O_7_^+^	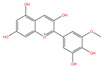	101	0.0087	0.3735	2.6768

**Table 2:** The top 10 core targets.

Number	Target Name	Degree	BC	CC	ASPL
1	BCL2	56	0.04368725	0.81818182	1.22222222
2	CASP3	56	0.04635699	0.81818182	1.22222222
3	NFKB1	54	0.03216106	0.8	1.25
4	MTOR	54	0.052009	0.8	1.25
5	JUN	53	0.03213428	0.79120879	1.26388889
6	TNF	53	0.03952339	0.79120879	1.26388889
7	HSP90AA1	51	0.03260088	0.77419355	1.29166667
8	EGFR	50	0.03866376	0.76595745	1.30555556
9	HIF1A	50	0.03640685	0.76595745	1.30555556
10	MAPK3	49	0.05609154	0.75789474	1.31944444

**Table 3:** QX feature map datasheet.

Marker Ingredients	Retention Time (min)	Peak Area	Relative Retention Time	Relative Peak Area
Amygdalin	24.373	412935	0.2501	0.0251
Puerarin	32.883	869521	0.3375	0.0529
Baicalin	97.435	16427378	1.0000	1.0000
Phillyrin	103.697	201714	1.0643	0.0123
Wogonoside	119.764	2860469	1.2292	0.1741
Baicalein	134.34	188517	1.3788	0.0115
Wogonin	157.575	252499	1.6172	0.0154

**Table 4:** The affinity value of the main ligands with receptors (*n* = 40).

Compound	Affinity kcal/mol	Protein	BCL2	CASP3	NFKB1	MTOR
(2R)-7-hydroxy-5-methoxy-2-phenylchroman-4-one			−5.17	−6.26	−4.77	−5.63
Quercetin			−5.58	−5.59	−5.30	−5.61
Dehydrodiisoeugenol			−6.01	−6.06	−6.06	−5.38
Amygdalin			−6.97	−6.56	−7.06	−5.99
Puerarin			−6.69	−6.50	−5.85	−5.80
Baicalin			−6.85	−6.52	−6.75	−5.85
Phillyrin			−7.56	−7.15	−7.09	−7.15
Wogonoside			−7.21	−6.94	−6.49	−6.36
Baicalein			−5.13	−5.55	/	−5.39
Wogonin			−5.33	−5.64	−5.01	−5.36

**Table 5:** The details of the medicinal materials of Qingxing granules.

Number	Herb Name Chinese Spelling	English Name	Latin Name	Plant Part	Weight Percentage
1	Qing Hao	Artemisiae Annuae Herba	*Artemisia annua* L.	Aboveground	10.42%
2	Ku Xingren	Armeniacae Semen Amarum	*Prunus armeniaca* L. var. ansu Maxim.	Seed	10.42%
3	Chai Hu	Bupleuri Radix	*Bupleurum chinense* DC.	Root	10.42%
4	Huang Qin	Scutellariae Radix	*Scutellaria baicalensis* Georgi	Root	10.42%
5	Lian Qiao	Forsythiae Fructus	*Forsythia suspensa* (Thunb.) Vahl	Fructus	10.42%
6	Ge Gen	Puerariae Lobatae Radix	*Pueraria lobata* (Willd.) Ohwi	Root	10.42%
7	Hua Shi	Talcum	Talcum	Ore	10.42%
8	Sang Ye	Mori Folium	*Morus alba* L.	Leaf	10.42%
9	Dou Kou	Amomi Fructus Rotundus	Amomum kravanh Pierre ex Gagnep.	Fructus	6.25%
10	Fu Ling	Poria	Poria Cocos (Schw.) Wolf	Sclerotium	10.42%

**Table 6:** Qingxing granules mobile phase gradient.

Time (min)	Mobile Phase A (%)	Mobile Phase B (%)
0~10	5%	95%
10~15	5~10%	95~90%
15~55	10~12%	90~88%
55~60	12~16%	88~84%
60~80	16~18%	84~82%
80~100	18~22%	82~78%
100~140	22~26%	78~74%
140~160	26~56%	74~44%
160~170	56~90%	44~10%
170~195	90~5%	10~95%

## Data Availability

Data are contained within the article and Supplementary Material.

## Supplementary Materials

The following supporting information can be downloaded at: https://www.mdpi.com/article/10.3390/ph17060731/s1, Figure S1: Drug Toxicity of Qingxing Granules on MDCK Cells; Figure S2: Qingxing Granules Inhibit IAV NP mRNA Expression in A549 Cells; Table S1: Design sequences for each primer.
