# Supplementary material for: Exploring the Underlying Mechanisms of Qingxing Granules Treating H1N1 Influenza Based on Network Pharmacology and Experimental Validation
Source: Pharmaceuticals (Basel). 2024 Jun 5;17(6):731. doi: 10.3390/ph17060731 (PMC11206762; doi:10.3390/ph17060731)
Supplement: Supplementary file 1 [file pharmaceuticals-17-00731-s001.zip › pharmaceuticals-3007874-supplementary.pdf]

In vitro experiments with Qingxing granules

1. Results

1.1 *Qingxing granules Drug Cytotoxicity Assay (MTT Method)*

Using the MTT assay to determine the toxicity of green apricot granules on MDCK cells, the results showed that the CC50 of green apricot granules on MDCK cells is 10.49 mg/mL. When the drug concentration is less than 5.78 mg/mL, the cell viability is greater than 80% (see Figure A1). Therefore, this concentration was used as the highest concentration for subsequent experiments.

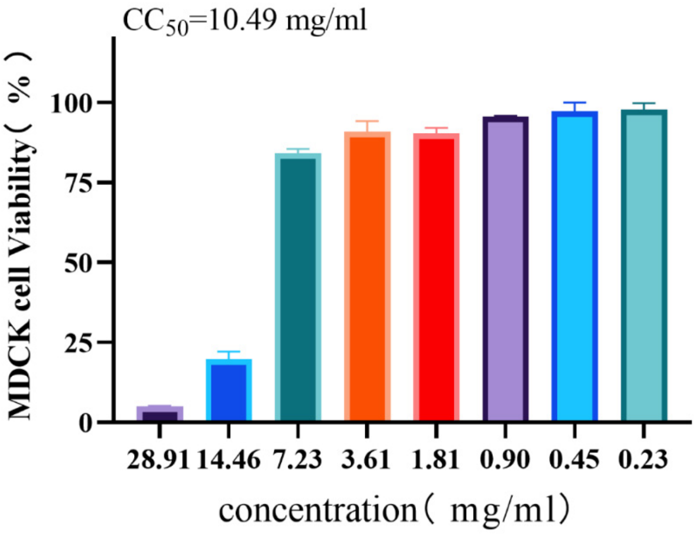

Figure S1. Drug Toxicity of Qingxing Granules on MDCK Cells (n=3)

1.2 *RT-qPCR*

The viral nucleoprotein NP gene was detected using RT-qPCR. The diluted viral solution (MOI = 0.05) was incubated with A549 cells for 1 hour, after which the viral solution was discarded and replaced with a specific concentration of QX blank 1640 medium. After further cultivation for 24 hours, RNA was extracted. The results showed that compared to the control group, QX could inhibit and reduce the expression of the NP gene in A549 cells. The inhibitory effect increased with the concentration, showing a concentration-dependent pattern, with statistically significant differences.

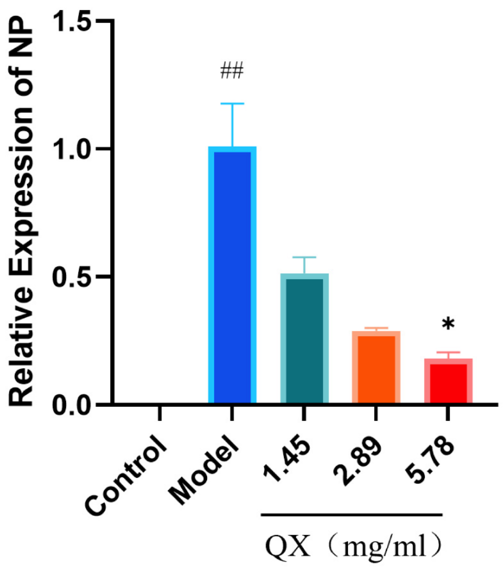

Figure S2. Qingxing Granules Inhibit IAV NP mRNA Expression in A549 Cells (n=3)

2. Discussion and conclusion

In vitro experiments with Qingxing granules against Influenza A H1N1 virus demonstrate that Qing Xing granules can significantly reduce the areas of cytopathic effect caused by Influenza A Virus (IAV) infection, and significantly lower the load of IAV progeny virus. Additionally, Qingxing granules can significantly inhibit the expression of the NP gene in the human non-small cell lung cancer cell line (adenocarcinomic human alveolar basal epithelial A549 cells), showing a dose-response relationship. This indicates that Qingxing granules can inhibit viral replication and the release of viral particles.

3. Materials and Methods

3.1 *Cells and Virus*

Madin-Darby canine kidney Cells (MDCK cells), Human Non-Small Cell Lung Cancer Cells (A549 cells); Influenza virus A/WSN/33(H1N1), A/PR/8(H1N1), provided by the Guangdong Provincial Key Laboratory of New Drug Screening, Southern Medical University.

3.2 *experimental drugs and reagents*

Qingxing granules（Batch Number: 201221）, Source as in the main text.

Cell RNA Extraction Kit: Fortis Bio, Catalog Number: RE03113, Batch Number: R220701;

Animal RNA Extraction Kit: Fortis Bio, Catalog Number: B03023, Batch Number: R210301;

GAPDH Primary Antibody: Fdbio Science, Catalog Number: FD0068, Batch Number: 20220310;

IAV NP Primary Antibody: GeneTex, Catalog Number: GTX636282, Batch Number: 42662;

Reverse Transcriptase: Takara, Catalog Number: RR0362-1, Batch Number: ALE1704A;

Fluorescent Quantitative PCR Enzyme: Evd Bio, Catalog Number: QE006-2, Batch Number: 20220604;

3.2 *Drug Cytotoxicity Assay (MTT)*

Take MDCK cells in good condition during the logarithmic growth phase, suspend them to 1×105 cells/mL, and seed 100 μL/mL per well in a 96-well plate, incubating overnight at 37°C in a 5% CO2 incubator. The next day, discard the culture medium, wash the cells with PBS, and add 100 μL/well of the test drug Qing Xing granules diluted in a gradient (diluted twice with blank DMEM culture medium), with three replicate wells per group. After continuing to incubate the plates in the incubator for 48 hours, discard the supernatant, wash the cells with PBS, and add 200 μL/well of MTT working solution (0.5 mg/mL). Continue incubating for 4 hours, carefully remove the supernatant, and add 150 μL/well of DMSO. After shaking at room temperature for 10 minutes, measure the

absorbance at OD=570 nm with a full-wavelength fluorescence microplate reader (LightCycler480, Roche, Switzerland) and calculate the drug's 50% cytotoxic concentration (CC50).

3.3 *Expression of viral nucleoprotein (NP) at the RNA level*

Refer to section 2.0 for cell treatment methods, discard viral supernatant and extract cellular RNA. Extract RNA following the procedure of the RNA Extraction Kit (#RE03113) (FOREGENE, China). The expression of NP after treatment with Qing Xing granules in cells was detected by RT-qPCR. The design of each mRNA primer is shown in Table A1.

Table S1. Design sequences for each primer

Target gene	Primer sequence（5’-3’）
NP-Forward	TGAGTGCAGACCGTGCTAAA
NP-Reverse	TCAAGTGAGAGAGAGCCGGA
GAPDH-Forward	AGGGCAATGCCAGCCCCAGCG
GAPDH-Reverse	AGGCGTCGGAGGGCCCCCTC
